# Supplementary material for: Contrast Volume-to-Estimated Glomerular Filtration Rate Ratio as a Predictor of Short-Term Outcomes Following Transcatheter Aortic Valve Implantation
Source: J Clin Med. 2024 May 17;13(10):2971. doi: 10.3390/jcm13102971 (PMC11122551; doi:10.3390/jcm13102971)
Supplement: Supplementary file 1 [file jcm-13-02971-s001.zip › jcm-2782829-supplementary.pdf]

Supplementary Material

Table S1. Baseline and procedural characteristics of study participants by AKI.

	**No AKI (n = 978)**	**AKI (n = 76)**	*** *p*-value**
Age, years	84 (79–88)	84 (78–87)	0.460
Female	486 (50)	33 (43)	0.292
Urgent	214 (22)	15 (20)	0.662
Diabetes	233 (24)	29 (38)	**0.005**
Hypertension	760 (78)	58 (76)	0.779
Previous MI	156 (16)	17 (22)	0.146
Prior revascularisation	155 (16)	13 (17)	0.773
SAVR history	24 (2.5)	1 (1.3)	>0.999
Previous stroke/TIA	161 (16)	15 (20)	0.461
PVD	69 (7.1)	8 (11)	0.263
Chronic pulmonary disease	233 (24)	22 (29)	0.315
Indication (AS or AR), AS	962 (98)	73 (96)	0.152
NYHA ≥ 3	601 (62)	49 (65)	0.519
Significant LMS disease	71 (7.6)	7 (9.9)	0.497
Significant non-LMS CAD	384 (41)	43 (61)	**0.001**
≥Moderate MR	652 (69)	46 (66)	0.568
LVEF, %			0.319
≥50	195 (20)	20 (27)	
30–50	195 (20)	16 (21)	
<30	583 (60)	39 (52)	
AV mean gradient, mmHg	42 (35–51)	40 (32–46)	**0.016**
AV peak gradient, mmHg	72 (61–85)	67 (54–77)	**0.021**
AV area, cm^2	0.70 (0.60–0.80)	0.70 (0.60–0.90)	0.172
PA systolic pressure, mmHg	36 (30–47)	36 (30–45)	0.879
Contrast volume used, ml	117 (98–150)	120 (100–156)	0.456
eGFR, ml/min/1.73 m^2			0.131
≥60	467 (48)	29 (38)	
30–59	461 (47)	45 (59)	
<30	50 (5.1)	2 (2.6)	
Euroscore II	3.00 (1.84–4.72)	4.17 (2.24–6.19)	**0.025**
GA	56 (5.7)	8 (11)	0.127
Procedural BAV	147 (15)	11 (14)	0.890
Contrast volume used, ml	117 (98–150)	120 (100–156)	0.456
TAVI delivery, femoral	952 (98)	73 (96)	0.249
Balloon-expanding valve	862 (88)	64 (84)	0.276
Successful valve deployment	926 (95)	74 (97)	0.576
CV/eGFR	2.14 (1.56–2.94)	2.32 (1.62–3.54)	0.060

Abbreviations: CV/eGFR—contrast volume administered normalised to estimated glomerular filtration rate, MI—myocardial infarction, SAVR—surgical aortic valve replacement, TIA—transient ischaemic attack, PVD—peripheral vascular disease, AS—aortic stenosis, AR—aortic regurgitation, NYHA—New York Heart Association, LMS—left main stem, CAD—coronary artery disease, MR—mitral regurgitation, LVEF—left ventricular ejection fraction, AV—aortic valve, PA—pulmonary artery, eGFR—estimated glomerular filtration rate, GA—general anaesthesia, BAV—bicuspid aortic valve, TAVI—transcatheter aortic valve implantation. Data are presented as medians (interquartile range) or number (percentage). * *p*-value based on chi-squared or Mann–Whitney U test.
